# CHIVA to treat saphenous vein insufficiency in chronic venous disease: characteristics and results

**DOI:** 10.1590/1677-5449.009918

**Published:** 2019-01-30

**Authors:** Felipe Puricelli Faccini, Stefano Ermini, Claude Franceschi

**Affiliations:** 1 Cirurgia Vascular, Hospital Moinhos de Vento, Porto Alegre, RS, Brasil.; 2 Instituto de Cardiologia, Porto Alegre, RS, Brasil.; 3 Veneinforma, Grassina, Florence, Italy.; 4 Centre Marie Thérèse, Hôpital Saint Joseph Paris, Paris, France.; 5 Hôpital Salpêtrière, Paris, France.

**Keywords:** CHIVA, saphenous sparing, local anesthesia, varicose vein, chronic venous disease, CHIVA, preservação safena, anestesia local, varizes, doença venosa crônica

## Abstract

There is considerable debate in the literature with relation to the best method to treat patients with chronic venous disease (CVD). CHIVA is an office-based treatment for varicose veins performed under local anesthesia. The aim of the technique is to lower transmural pressure in the superficial venous system and avoid destruction of veins. Recurrence of varicosities, nerve damage, bruising and suboptimal aesthetic results are common to all treatments for the disease. This paper evaluates and discusses the characteristics and results of the CHIVA technique. We conclude that CHIVA is a viable alternative to common procedures that is associated with less bruising, nerve damage, and recurrence than stripping saphenectomy. The main advantages are preservation of the saphenous vein, local anesthesia, low recurrence rates, low cost, low pain, and no nerve damage. The major disadvantages are the learning curve and the need to train the team in venous hemodynamics.

## Resumo

Existe uma grande discussão na literatura sobre o tratamento da doença venosa crônica (DVC). A cura conservadora e hemodinâmica da insuficiência venosa em ambulatório (CHIVA) consiste no tratamento ambulatorial de varizes sob anestesia local. O objetivo da técnica é diminuir a pressão transmural no sistema venoso superficial para evitar a destruição das veias, incluindo as veias safenas. Recorrência de varizes, lesão de nervos, hematomas e resultado estético abaixo do ideal são uma constante em todos tratamentos de varizes. O objetivo desta revisão é avaliar e discutir a técnica CHIVA quanto a suas características e resultados. A CHIVA é uma alternativa válida frente aos outros procedimentos, apresentando menos hematomas, recorrência e lesão nervosa que a safenectomia. Preservação da veia safena, anestesia local, baixa taxa de recorrências, baixo custo, pouca dor e ausência de lesões nervosas são as principais vantagens. A longa curva de aprendizado para treinar a equipe em hemodinâmica venosa é a principal desvantagem.

## INTRODUCTION

 CHIVA is the French acronym for “Cure conservatrice et Hemodynamique de l’Insuffisance Veineuse en Ambulatoire” (Conservative and Hemodynamic treatment of Venous Insufficiency in the Office). It is a saphenous-sparing therapeutic approach to lower limb chronic venous disease (CVD) based on hemodynamic concepts proposed by Claude Franceschi in 1988. 1 ^-^ 6 The rationale behind this hemodynamic approach to treating the disease is that increased transmural pressure (TMP) is responsible for progression of the signs and symptoms of CVD, such as varicosities, edema, pain, itching, dermatitis and ulcers. Transmural pressure is elevated in superficial venous disease because of the higher hydrodynamic pressure caused by absence of orthodynamic pressure fractionating and presence of closed shunts. 7

**Figure 1:**
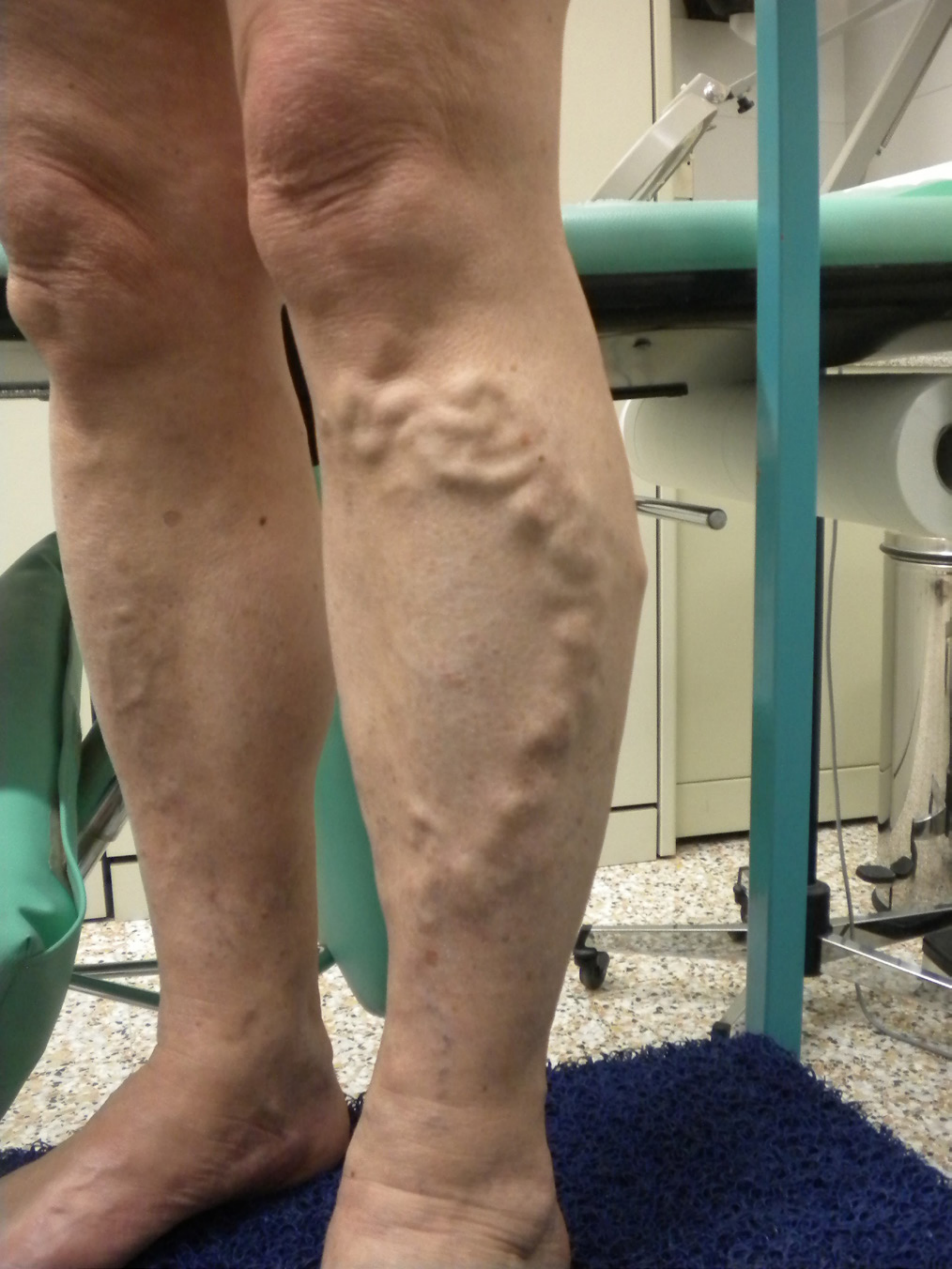
Preoperative image of a CHIVA patient.

**Figure 2:**
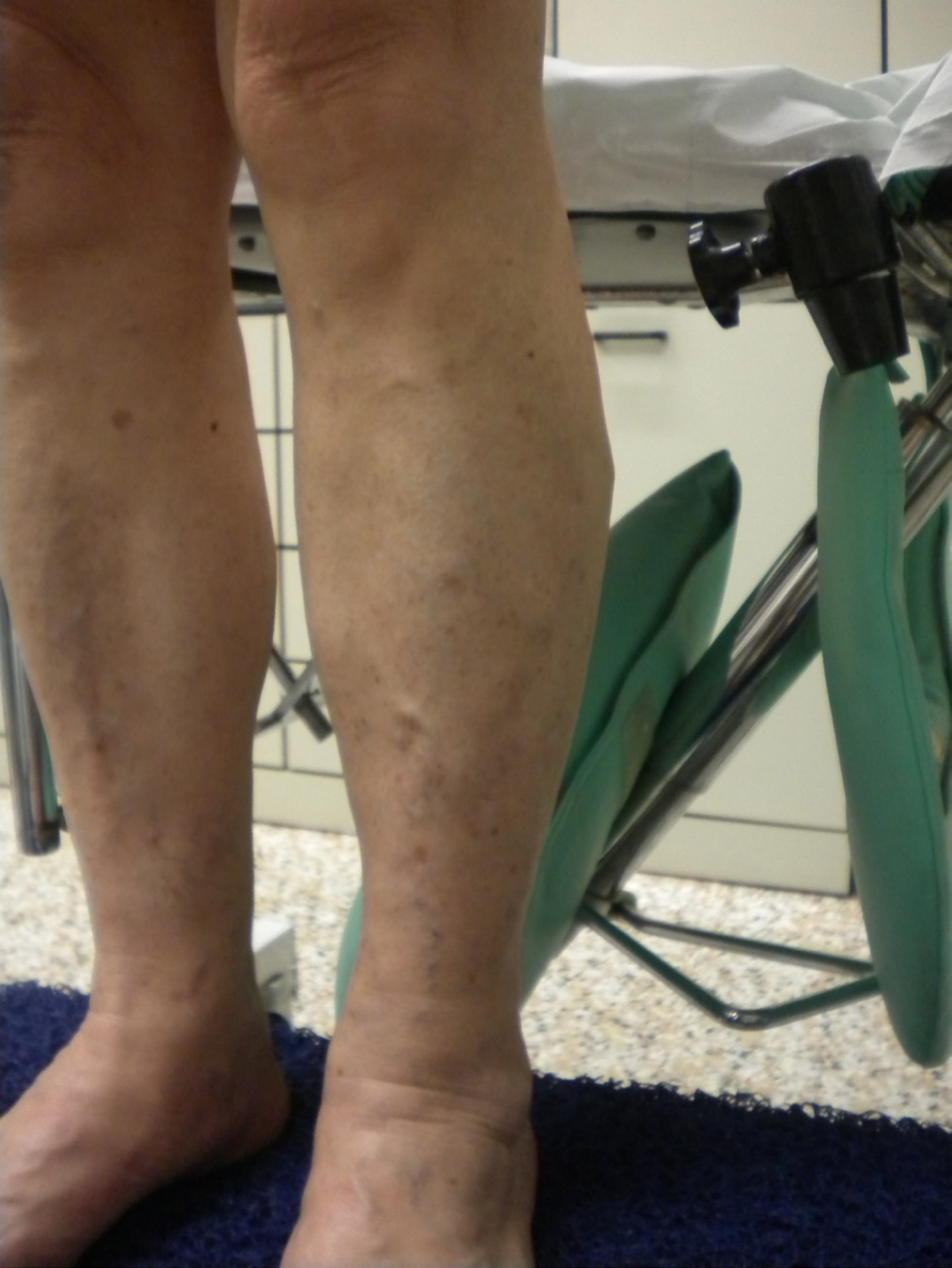
Postoperative image after CHIVA - No phlebectomy, sclerotherapy, laser or any other treatment was performed. Veins disappear due to lower transmural pressure.

 The CHIVA strategy aims to restore near-normal physiological flow with no destruction or ablation of the veins involved. The mainstay of this approach is a correct hemodynamic evaluation. A complete duplex scan is performed to correctly determine the source of pressure overloads. 8 The strategy uses ligatures targeted to interrupt escape points and fractionate hydrostatic pressure. The number and position of these ligatures depend on duplex scan findings and every operation is tailored to each patient’s reflux pattern. No phlebectomies are performed and reduction of TMP causes the varicose veins to reduce in size, as shown in preoperative and postoperative images ( Figures 1 and 2 ). 

 The procedure is performed under local anesthesia and can be conducted in the office with immediate patient discharge. The great and small saphenous veins are left in place and are available in the future for bypass surgery and to channel the flow of varicose recurrence, if this occurs. All collateral veins are preserved and return to their normal size in a few months after the hemodynamic result is achieved. This is particularly interesting because the postoperative period after phlebectomy can be painful and may be complicated by pigmentations and red telangiectasias that are difficult to treat. Additionally, studies suggest that extensive resection of veins may result in higher recurrence rates over the long term. 9 Recently, saphenous sparing techniques have been increasingly discussed in the literature and are being considered as a promising approach to treatment of CVD patients. 10

 The objective of this review article is to briefly describe the CHIVA technique and present the results of a technique that is a possible cost-effective alternative to the usual venous ablative/resective procedures. 

## DISCUSSION

 Several different procedures for treatment of varicose veins are possible and offer good results. The CHIVA strategy is based on venous system hemodynamics and aims to maintain the venous system in place while correcting imbalances created by shunts between the deep and superficial venous systems. 3 The main characteristics of the procedure are: 1) local anesthesia; 2) day-clinic surgery; 3) immediate return to activities; 4) low pain scores; 5) avoidance of removal of collaterals causing fewer skin marks; and 6) preservation of the saphenous trunks in place for future bypass use and to receive flow from any new escape points or recurrence (making them easier to treat). 

### The CHIVA strategy: basic principles

**Figure 3:**
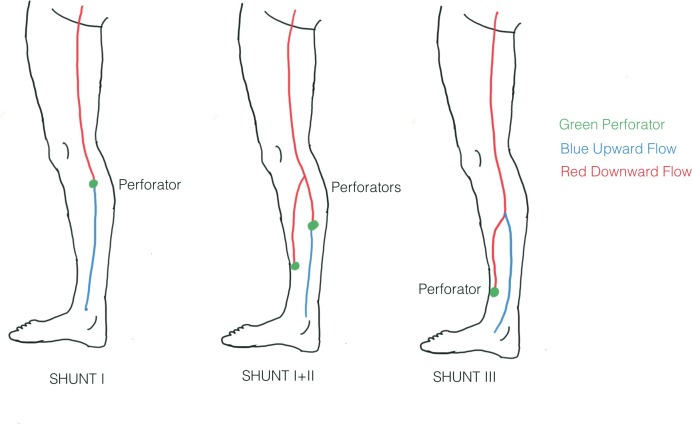
Types of Shunts. Shunt I, reentry straight from the saphenous vein. Shunt I + II, reentry from the saphenous vein below the collateral and the collateral itself. Shunt III, no reentry from the saphenous vein, only from the collateral.

 The objective of treatment is to maintain the saphenous vein and collaterals draining to the deep venous system, independently of the direction of flow. In some cases, the saphenous vein will recover and the flow will be directed upwards. However, in other cases, in which the vein is too large or the saphenofemoral junction is the primary source of reflux, flow will be directed downwards and reenter the deep system via the perforators. This downward flow is not pathogenic and is associated with venous system stability and good long term results. The technical approach employed in CHIVA depends on the shunts identified in each patient. Basically, the escape point (start of reflux) should be treated, usually by ligation. The reentry point is preserved (where reflux enters the deep system after its superficial course). Collaterals found along the course of reflux that might maintain or create reflux should be also interrupted. Collaterals and saphenous veins should not be left without reentry points because of the risk of thrombophlebitis. For example, in a type I or I+II shunt, the refluxing saphenous vein has direct drainage to a perforator, as have the collaterals involved. On the other hand, a type III shunt has no direct saphenous flow drainage to a perforator; rather, the sequence is saphenous vein – collateral – perforator ( Figure 3 ). In the first case (type I+II shunt), ligation of the collateral will reduce diastolic reflux and TMP in the saphenous vein. In the second case (type III shunt) ligation of the escape point and disconnection of the tributary will result in absence of flow in the saphenous trunk. This can cause saphenous thrombosis that will be recanalized as soon as a new reentry point develops. Further strategy possibilities, such as devalvulation, use of tributary perforators or CHIVA in 2 steps (CHIVA 2) offer adequate treatment for such cases. The 2-step CHIVA strategy consists of an initial ligature of a collateral, leaving the saphenous vein untouched in the first procedure. Disconnection of the tributary in a type III shunt eliminates the centrifugal flow, decreasing the saphenous caliber due to the reduced flow rate. The energy of a shunt from the escape point is not always stable or predictable and centrifugal flow in the GSV trunk can reappear if a new reentry point occurs. In this case, a second CHIVA step is performed to treat the escape point. This second procedure is planned in advance and is not considered a reoperation, but rather the completion of the first. This is commonplace with the CHIVA approach, but is often erroneously considered a reoperation by surgeons unfamiliar with the technique. 11

### Characteristics of CHIVA

#### Nerve damage

 One of the biggest advantages of CHIVA compared to other venous procedure modalities is the absence of nerve damage, particularly in the modern context in which nerve damage and malpractice claims are becoming a common problem. During a CHIVA procedure, performed under local anesthesia, the patient warns the surgeon if the sural or saphenous nerves are touched, which they cannot do with general, axial, or tumescent anesthesia, because the nerve or response are blocked. The nerve is therefore susceptible to damage by the mechanical or burning energies used in most common procedures such as vein stripping and thermal ablation. Two previously published randomized clinical trials comparing stripping to CHIVA found evidence of no cases of nerve damage in 286 CHIVA procedures compared to 26 nerve damage cases in 383 (6.7%) stripping procedures. 12 ^,^ 13 A study of litigation claims after vascular surgery showed that nerve damage is responsible for nearly a third of malpractice claims that are successful in the courts after venous operations. This British study showed a 61% success rate of malpractice claims related to varicose veins operations. 14 Mean compensation for damage was €100,000 and in some cases awards were higher. In Brazil, there is no transparency in the courts and the healthcare system per se (both insurance and public) is usually left out of these trials. We do not have clear data on success rates or a direct evaluation of the financial burden these trials impose on doctors. Current knowledge suggests that compensation awards are lower than in the British study, but the completely free justice system (without no cost to the plaintiff in the case of an unsuccessful claim) makes the number of malpractice suits much higher than in England. We consider that the CHIVA technique causes near zero nerve damage and may be advantageous for avoiding such mishaps. 

#### Future use of the saphenous vein

 The importance of preservation of the saphenous veins in venous operations is a matter of great debate. Possible advantages include maintaining the vein for further use in bypass surgeries, reducing surgical trauma to prevent remodeling, and retaining the saphenous trunk to receive flow in case of a recurrence. With regard to bypass surgery, use of the saphenous vein in both coronary and peripheral bypass surgery is well established in the literature. 15 ^,^ 16 The prevalence of coronary disease varies according to population. In Brazil, from 2005 to 2007, a total of 63272 coronary artery bypasses were performed, equating to a total of 1 operation for every 2900 inhabitants in the general population. 17 Another study showed that in Rome one coronary bypass was performed for every 1424 inhabitants over the age of 35 years during the late nineties. 18 Recent studies show that harvesting the saphenous vein using no touch techniques is reliable and has a long term patency comparable to the internal thoracic artery. 18 ^,^ 19 Concerning peripheral bypass for limb ischemia, a national study in the United States found that 1.6% of patients with peripheral artery disease underwent a peripheral bypass. 19 Surgical removal or ablation of the saphenous vein may decrease the likelihood of treatment success in patients needing such bypasses. 20

 Patients who present with deep vein thrombosis or leg trauma after a venous operation may need the great saphenous vein for adequate venous return. If the vein has not been destroyed previously, a vicarious shunt may form in such patients, improving symptoms. 

#### Recurrence of varicose veins

 Recurrence of varicosities is a constant concern in patients undergoing venous operations and places considerable burden on patients over the long term. There are recent biochemical and clinical studies suggesting that excessive venous resection may cause more recurrence. Biochemical and animal studies show that increases in the pressure on veins and chronic shear stress on the vein wall are linked to venous remodeling and may lead to recurrence. 21 ^,^ 22 Animal studies have shown that transcription factor activator protein 1 (AP-1) appears to be a prerequisite for venous remodeling/proliferation and MMP-2 (matrix metalloproteinase) expression. MMP-2 expression and venous proliferation are stimulated by sudden interruption of the ear vein in rats. 21 ^,^ 22 Additionally, a clinical study showed that ligation of all junctional saphenous tributaries is associated with a higher risk of varicose vein recurrence. This study compared recurrence in two groups after high ligation of the saphenofemoral junction, with or without ligation of all tributaries. The group that had all tributaries ligated had a sevenfold increase in recurrence. 9 These data suggest that an approach with less resection may help reduce recurrence. Indeed, in the CHIVA strategy, the approach to SFJ incompetence consists of sectioning/ligating the saphenous arch at the common femoral vein junction, preserving the collaterals draining into the saphenous arch. Furthermore, we should remember that chronic venous disease is a lifelong disease and recurrence is a constant problem. The longer the follow-up periods of clinical trials, the better the knowledge they offer about the long-term results. 

 With regard to the results and safety of CHIVA, there are several studies and some randomized clinical trials (RCT) comparing CHIVA with stripping/compression in different subtypes of patients. Zamboni studied severe cases and ulcer patients in a RCT comparing healing and recurrence of ulcers in two groups of patients (CHIVA and compression therapy). 23 The study showed that CHIVA had a higher healing rate than compression (100% versus 96%) and less ulcer recurrence over a 3-year period (9% versus 38%). The study showed that CHIVA is a safe and effective treatment for venous ulcers with better results than compression therapy. 

 There are also some RCTs comparing CHIVA to stripping for chronic venous disease without ulcers and several studies confirming its efficacy and reporting good results. 5 ^,^ 24 ^,^ 25 Iborra-Ortega et al. 12 published the results of a randomized trial comparing CHIVA with stripping in 100 patients over a 5-year follow-up period. This study found no difference between the CHIVA and stripping groups in recurrence, reoperation, or aesthetic results. Carandina et al. 26 randomized 150 patients and followed stripping and CHIVA groups for up to 10 years. This study found a twofold higher recurrence rate in the stripping group. It also showed that recurrence was significantly higher after stripping (in both stripping groups; a group in which veins were marked clinically and a group in which veins were marked using duplex scanning) than in the CHIVA group (odds ratio 2.64 and 2.01 respectively). In an RCT, Parés et al. 13 showed that recurrence up to ten years’ follow-up was 31.1% for the CHIVA method, compared to 50.3% for stripping. The RCTs investigating CHIVA were open and did not blind participants or personnel to which group participants were assigned to, because it is easy to recognize the type of operation, since anesthesia, incisions, and duplex findings make identification of groups possible. 

 A Cochrane systematic review including clinical trials evaluating CHIVA compared to stripping showed significantly less nerve damage, fewer bruises, and less recurrence. 27 The results favored the CHIVA approach, although the review authors suggested further studies are needed to corroborate findings. The authors considered the lack of blinding of patients and personnel to be a possible source of bias. They recommended further trials using quality of life endpoints and comparing CHIVA to other vein surgery modalities. 

 There are several treatment alternatives for recurrent varicose veins and a high incidence of recurrence is commonplace. A Brazilian study presented good results after open correction for groin recurrence, although complications such as skin infection, lymphedema, and recurrence are a matter of concern. 28 Sclerotherapy has a good success rate and is being increasingly used worldwide, but deep vein thrombosis and recurrence are possible complications. Recurrence is common after saphenous stripping and tends to increase with time elapsed after the operation. Recurrence after endovenous laser ablation (EVLA) of the saphenous vein seems to be similar to the rate of recurrence after stripping. Rasmussen et al. 29 showed that clinical recurrence rates after EVLA and stripping were 46.6% and 54.7% respectively and reoperation rates were 38.6% and 37.7% at 2 years. In this study, there were no statistical differences in recurrence or reoperations between EVLA and stripping. There are few published RCTs comparing recurrence after CHIVA and EVLA, although Chan et al. 30 showed that CHIVA patients exhibited less pain and less need for sclerotherapy after operations than EVLA patients. The issue of recurrence is also a problem after foam treatment and no long term results are available for adequate comparison with other techniques. 31 ^,^ 32 These data suggest that there is no conclusion concerning which is the best method to avoid recurrence. There is one RCT investigating the CHIVA technique with 10 years’ follow-up in which it was associated with less recurrence than the stripping technique, making it a good option for treating patients with saphenous vein insufficiency. Gloviczki et al. 33 published a guideline for chronic venous disease and considered the results of preservation of the saphenous vein with CHIVA. The conclusion was that the results were better than compression for preventing ulcer recurrence and at least equivalent to stripping of varicose veins. 

#### Bruises and aesthetic results

 Most patients who undergo varicose vein operations have high expectations and skin marks, bruises, and brown spots are common patient complaints. The aesthetic results of CHIVA have been compared to those of stripping operations. Parés et al. 13 found significantly fewer bruising marks after CHIVA (45%) than after saphenous vein stripping (76%). Aesthetic improvements were assessed in terms of participants’ opinions in two trials and no significant differences were found. 12 ^,^ 26 Aesthetic improvements as assessed by the investigator were recorded in one trial and no significant difference was found between CHIVA and stripping. 12 Parés et al. 13 reported a significant difference in favor of CHIVA in relation to postoperative bruises, with 240 out of 334 patients (71%) exhibiting bruising after stripping compared to 76 out of 167 (45%) CHIVA patients. This is probably because most veins are left in place and less subcutaneous blood remains to stain the skin. We conclude that the CHIVA technique causes less bruising than the stripping technique, probably because no phlebectomies are conducted. No significant differences in patients’ aesthetic impressions were observed. 

#### Learning curve

 The CHIVA technique demands that the surgeon has expertise in venous hemodynamics. The learning curve is long and is extremely important to achieve good results. Surgeons who are not specifically skilled in CHIVA may have high superficial thrombophlebitis incidence rates, 34 because of excessive ligature of draining collaterals or failure to recognize disproportionate calibers in different regions of the saphenous vein. The incidence of this complication diminishes with training, experience, and correct planning of the procedure. For example, in one RCT, Parés et al., 13 observed a 1.3% phlebitis rate in the CHIVA group and there was no significant difference between this group and the stripping patients. In our experience, the learning curve is long and appropriate duplex scanning skills that are not covered in the usual teaching units are required. The incidence of superficial phlebitis at our clinic (Brazilian author) is below 2% of cases, all cases were asymptomatic and diagnosed by duplex scan. No case of symptomatic phlebitis has been observed to date. A trial comparing CHIVA and stripping found that CHIVA performed by experienced surgeons had a significantly lower recurrence rate and better results than stripping, but CHIVA patients operated by non-specialized surgeons had worse results. 35 The authors point out that the several different possibilities in the CHIVA technique make it less repeatable than stripping and demand more training. Gloviczki et al. 33 concluded that CHIVA is a complex approach and that high levels of training and experience are needed to achieve the results reported by RCTs. Venous interventionists willing to learn this approach require considerable education. 

 Postoperative GSV thrombosis is due to the absence of centrifugal flow and occurs more commonly after treatment of type III shunts. This phlebitis is due to a hemodynamic cause, rather than endothelial trauma, as occurs in foam sclerotherapy or endovenous ablation. The difference is that this thrombosis will recanalize as soon as a new efficient reentry point develops, generally from 1 to 6 months after surgery. With the CHIVA strategy, GSV thrombosis is an event of low significance and cannot be considered a failure of the technique, since the majority of thrombosed GSVs regain flow in a few months and cause no symptoms. This is common to other saphenous-sparing techniques. For example, Ferracani et al. 36 presented a saphenous-sparing laser remodeling procedure associated with a 10.5% rate of saphenous thrombosis; half of these patients had spontaneous recanalization within a few weeks. 

#### Postoperative duplex scan

 Another important point that is pivotal to physicians performing duplex scans and surgeons who do not perform the CHIVA is the concept of postoperative reflux. The usual approach to reflux soon after stripping or EVLA is to consider this a treatment failure. The purpose of CHIVA is to keep the veins draining and refluxing veins might recover upward flow or continue to drain reflux. Many patients remain with a continuously draining reverse flow without a compartment change (the saphenous diameter decreases), classified as a type 0 shunt. 3 This is considered a good treatment result, fulfilling the ultimate objective of a CHIVA procedure. 

#### The cost of CHIVA

 The cost of CHIVA is low if compared to newer techniques that use expensive industry technology. This can be particularly advantageous in developing countries and for clinics/doctors that earn similar fees irrespective of the technique used. On the other hand, the method does not attract funding for clinical trials and publicity because the procedure does not involve expensive industry equipment. The CHIVA surgeon spends more time with the patient than with other techniques and must perform duplex scans to diagnose, mark the skin and follow-up the patient. The extra duplex examinations and additional surgeon time with the patient should be also considered. Zmudzinski et al. 37 published a trial showing good early results of CHIVA, but pointed to obstacles to disseminating the technique related to the need for detailed duplex scans, the existence of accessible alternatives such as EVLA/stripping, and insurance-related problems. Nevertheless, the study concluded that several investigators have achieved good results and suggested keeping an open mind with regard to CHIVA. 

## CONCLUSION

 CHIVA is a cost-effective method for treating CVD patients. The possible advantages are no nerve damage, preservation of the saphenous vein (both for bypass and to receive flow from recurrent varicosities), a low recurrence rate, local anesthesia, and reduced bruising. The main disadvantage is the long learning curve needed to master the technique. 
